# The Current Research of Spatial Cognitive Evaluation and Training With Brain–Computer Interface and Virtual Reality

**DOI:** 10.3389/fnins.2019.01439

**Published:** 2020-02-07

**Authors:** Yanhong Zhou, Dong Wen, Huibin Lu, Wang Yao, Yijun Liu, Wenbo Qian, Jingpeng Yuan

**Affiliations:** ^1^School of Information Science and Engineering, Yanshan University, Qinhuangdao, China; ^2^School of Mathematics and Information Science and Technology, Hebei Normal University of Science and Technology, Qinhuangdao, China; ^3^The Key Laboratory for Computer Virtual Technology and System Integration of Hebei Province, Yanshan University, Qinhuangdao, China; ^4^The Key Laboratory of Information Transmission and Signal Processing of Hebei Province, Yanshan University, Qinhuangdao, China; ^5^School of Science, Yanshan University, Qinhuangdao, China

**Keywords:** spatial cognition, training, evaluation, brain–computer interface, virtual reality

## Introduction

Spatial cognitive evaluation and training (SCET) is a rapidly growing research field (Chunyin et al., 2011) in cognitive study. SCET is also greatly significant in the diagnosis and rehabilitation of mild cognitive impairment (MCI), mainly because patients with MCI show symptoms of spatial cognitive impairment at an early stage (Allison et al., 2016; Laczó et al., 2016). For SCET, real-time and accurate quantification is the ultimate goal in evaluation (Lin et al., 2015); it is expected to have a strong sense of participation for the subjects in training, and the training content is closely related to their daily life (Bormans et al., 2016).

Virtual reality (VR) (Tu et al., 2017) and brain–computer interface (BCI) (Xu et al., 2013) are popular technologies in SCET. Training with VR meets experience and social needs of subjects and can be used as the main way of spatial cognitive training (SCT) (Serino et al., 2015; Bormans et al., 2016; Davis and Ohman, 2016; Migo et al., 2016; Tu et al., 2017; Zygouris et al., 2017). However, in these studies, it is difficult to understand the training effect in real time to the subjects and trainers, although they are very eager to observe the training effect timely so as to adjust the training state or program. BCI based on electroencephalogram (EEG) signals (Xu et al., 2018) is often used for real-time SCET and can be applied to real-time monitoring of brain activity on the premise of high temporal resolution (Lin et al., 2015; Han et al., 2017; Chen et al., 2018; Guevara et al., 2018; Pergher et al., 2018). Therefore, it is a good choice to combine BCI and VR (Lechner et al., 2014; Koo et al., 2015; De Tommaso et al., 2016; Donati et al., 2016; Vourvopoulos and I Badia, 2016) for SCET, and there are preliminarily applied studies (Bischof and Boulanger, 2003; Jaiswal et al., 2010; Kober and Neuper, 2011; Tarnanas et al., 2015), which suggested that BCI-VR was a recommendable method of SCET. However, this combination is still in its infancy, and more work needs to be done before conclusions can be confidently drawn.

This study will review the literature related to SCET with VR, BCI, and BCI-VR; discuss the potential advantages of BCI-VR in SCET and future problems to be solved; and put forward our opinions. It is expected that this analysis may provide valuable suggestions for the field of information technology in SCET.

This study used the Web of Science–Science Citation Index/Social Sciences Citation Index (WOS–SCI/SSCI) database, focused on the studies of BCI, VR, and BCI-VR in SCET. The following search keywords were used: “spatial cognitive evaluation (SCE)” or “spatial cognitive training (SCT)” in combination with “brain–computer interface (BCI)” or “virtual reality (VR).” The most recent search was conducted on March 21, 2019.

## Research Status of SCET With VR

It is a relatively independent cognitive factor for spatial cognitive ability (SCA), which can be improved by reasonable training (Chunyin et al., 2011). The water maze with VR, which has become the most classical experimental method in the field of SCET, was used to measure the SCA of rats (Morris, 1984). This method can be used to train the SCA of rats (Chunyin et al., 2011) and distinguish male and female participants (Astur et al., 1998). Currently, VR opens up a new way for the research of SCET and had been applied to some researches of SCET (Bormans et al., 2016; Tu et al., 2017), in which VR could help subjects to experience a strong sense of participation in training process, and the training environment with VR was not limited by the area.

In terms of SCE, VR is widely used in the evaluation of AD and MCI (Weniger et al., 2011). For AD patients, virtual hospital (Jiang and Li, 2007), virtual auditorium (Lange et al., 2007), virtual city (Zakzanis et al., 2009), virtual building (Cushman et al., 2008), and virtual environment of apartment (Davis and Ohman, 2016) can be used to evaluate the path finding and navigation ability. When implementing a virtual path learning task, preclinical AD showed disorder (Allison et al., 2016), and there are correlations between regional neurodegeneration of AD and ability of spatial terrain memory (Pengas et al., 2012). In addition, relocation tasks with VR can be used to assess the spatial memory of early AD (Shamsuddin et al., 2011, 2012; Caffo et al., 2012). For MCI patients, the missing ability to orientation in virtual space (Morganti et al., 2013) and virtual supermarket (Tu et al., 2015; Zygouris et al., 2017) can help us to screen them from all subjects (Zygouris et al., 2017). In addition, the virtual path navigation can be used to evaluate their visual spatial memory (Lesk et al., 2014), and virtual room location search can be used to detect their obstacles in spatial navigation (Serino et al., 2015).

In terms of SCT, there existed several VR games widely used by cognitive impairment patients. Virtual memory palaces game can be used to improve the quality of life and memory of AD (Bormans et al., 2016), virtual buildings navigation game can be applied to improve the driving skills and daily cognitive ability of AD patients (White and Moussavi, 2016), and the large virtual outdoor parks game can be used to train the navigation ability of patients with dementia (Flynn et al., 2003). In addition, other VR games can also be used to improve the spatial attention of cognitive impairment patients (Manera et al., 2016).

## Research State of SCET With the Change of BCI Signals

Previous studies have shown that spatial memory, spatial orientation, and navigation dominated by the hippocampus were important components of spatial cognition (Olton et al., 1979). They also have shown that the dynamic characteristics of EEG signals from the parietal lobe were directly related to the ability of spatial memory and navigation (Chiu et al., 2012). Therefore, it is feasible to explore the changes of SCA from the perspective of EEG signal analysis.

From the perspective of SCE, BCI signals are widely used in the evaluation of spatial navigation and memory. For spatial navigation, dynamic features of the EEG signal can respond to the change of spatial navigation (Lin et al., 2009, 2015). The navigation performance was related to the power modulation in theta or gamma frequency band (Bell, 2002; White et al., 2012; Chen et al., 2018), Especially, the phase reset of theta rhythm during spatial navigation caused the NT170 latency effect (Baker and Holroyd, 2013). Interestingly, female participants showed stronger theta oscillations during spatial navigation (Nishiyama et al., 2002). For spatial memory, the perception and action in the memory task are different by EEG network analysis (Protopapa et al., 2011). The power of EEG signals from AD patients on alpha and theta frequency bands was significantly higher relative to normal control when performing object-location memory tasks (Han et al., 2017). In addition, when the delay, peak amplitude, and root mean square of P300 act as the features in visual spatial memory paradigm, the classification accuracy could achieve up to 91.76% (Li et al., 2013).

From the perspective of SCT, almost all of the studies focused on the training of spatial memory, which can enhance the connection between nervous activity and the brain network in frontal, parietal, and occipital lobes of MCI patients (Hampstead et al., 2011). In addition, the Pearson correlation coefficient between the right prefrontal lobe regions decreased after spatial memory task, which indicated that the region had a higher participation in memory task (Plank et al., 2010; Guevara et al., 2018), Surprisingly, old people showed higher amplitude of P300 in the parietal lobe than the young on spatial memory training (Pergher et al., 2018),

Based on the above researches, BCI can provide support for objective data analysis in SCET and can effectively improve the scientific quantification performance of this research field, as well as the operability and repeatability of experiments.

## Research Status of SCET With BCI-VR

### Research Value of BCI-VR

In recent years, the combination of BCI and VR has provided practical benefits for training and evaluation (Achanccaray et al., 2017). In VR training, BCI can be used to analyze brain activity synchronously in real time (Martišius and Damaševičius, 2016). Meanwhile, VR can provide a situation similar to the daily life, in which the brain activity could be evaluated by EEG analysis (Alchalcabi et al., 2017). Therefore, the fusion of VR and BCI can solve the subjective and nonreal-time evaluation of training effect in the training process. Recently, BCI-VR has been successfully used in some application fields, including steady-state visual evoked potential (Lechner et al., 2014) and motor imagery (I Badia et al., 2012; Vourvopoulos and I Badia, 2016) for stroke rehabilitation, which can overcome the limitation of traditional monitor refresh frequency (Calore et al., 2014; Koo et al., 2015) and increase the engagement of subjects (Koo and Choi, 2015). In addition, it has other applications, such as motion imaging for paraplegic rehabilitation (Donati et al., 2016), P300 for cognitive training of normal control (De Tommaso et al., 2016), autism training (Amaral et al., 2017), and hyperactivity training (Rohani and Puthusserypady, 2015).

### Research Analysis of SCET With BCI-VR

**Figure 1:**
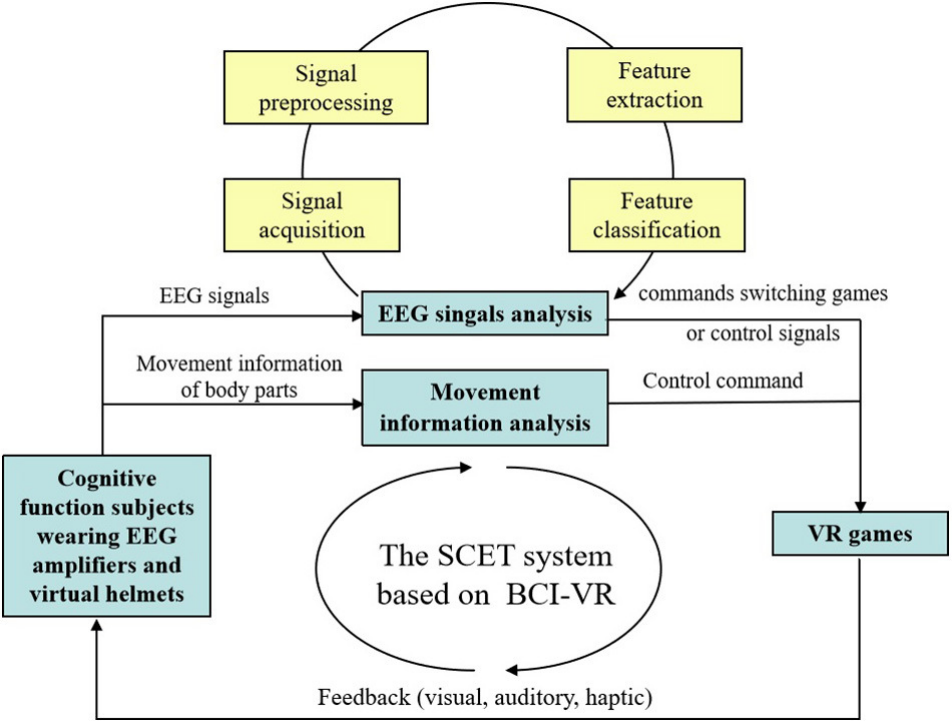
The schematic diagram for the system description of the SCET with BCI-VR.

At present, there are several studies that combine BCI and VR to exert their respective advantages in the field of SCET. For the spatial navigation, Bischof and Boulanger (2003) found that theta oscillation of EEG signals was related to the encoding and retrieval of spatial information when performing VR maze navigation tasks, and Silvia et al. compared the differences of EEG signals from male and female adults on theta frequency band with VR spatial navigation tasks (Kober and Neuper, 2011). For the spatial memory, Jaiswal et al. (2010) combined BCI with VR to observe differences in EEG activity during the coding and retrieval stages of spatial memory tasks. For other spatial task, Tarnanas et al. (2014a,b); Tarnanas et al. (2015) used VR day-out task to evaluate its predictive value of MCI. All of these studies have verified the rationality of the combination of BCI and VR in SCET research. Figure 1 showed the schematic diagram for the system description of the SCET with BCI-VR.

## Discussion

Many studies suggested that VR can be used to the SCET (Bormans et al., 2016; Manera et al., 2016; Tu et al., 2017; Zygouris et al., 2017) and changed the old way of SCE with neuropsychological scales, subjective judgments, qualitative description, and so on (Manera et al., 2016). VR technology has obvious advantages in immersive space experience and internal space visualization in the field of architectural design. However, VR is still unable to evaluate the effects of SCT in real time like BCI. Many studies also suggested that the change of BCI signals can be used to the SCET (Han et al., 2017; Chen et al., 2018; Guevara et al., 2018; Pergher et al., 2018). BCI can be used to analyze EEG signals in SCET effectively and improve the operability and repeatability of evaluation and training. However, BCI cannot supply an abundant environment for SCET compared to VR. Several studies in the field of SCET combined BCI and VR to exert their respective advantages (Jaiswal et al., 2010; Kober and Neuper, 2011; Tarnanas et al., 2015). Compared with BCI and VR used in SCET, BCI-VR not only can improve the effect of spatial cognition training but also can overcome the limitation of spatial cognition evaluation.

However, various challenges to be broken through currently exist in BCI-VR used in SCET. Firstly, effective interaction remains to be achieved in VR, so a new interaction model, which combines natural interaction in VR with BCI, needs to be studied. Secondly, wearing the wet electrode in BCI increases the experimental preparation time compared with the dry electrode in BCI. Currently, there is a lack of the dry electrode with high signal quality and good comfort. Therefore, the design and development of electrode in the future need to be strengthened. Thirdly, the SCT in the real environment requires a large space, but the range of activity scenes covered by existing VR equipment cannot meet this demand. Hence, the algorithm that can scale up or down the VR game scenes at will is very important.

## Conclusions

In conclusion, this study reviewed the recent literature of VR, BCI, and BCI-VR used in SCET. Although these studies obtained promising results, more work needs to be done before conclusions can be confidently drawn. It is suggested that BCI and VR should be deeply integrated in order to give full play to their respective technical advantages. The current technical bottlenecks of BCI and VR should be broken through to provide personalized, comfortable, and real-time technical support for creating an effective environment for SCET. Therefore, in the future, for the SCET, it will be necessary to not only solve the technical problems of BCI-VR but also to take patients as important subjects, design personalized and adaptive BCI-VR games, and train the patients. In addition, the training effect had better be evaluated and fed back in real time in order to meet the individual needs of patients.

## Author Contributions

HL, YZ, WY, and YL designed the work. YZ, DW, and HL wrote this manuscript. YZ, DW, WY, YL, WQ, and JY collected and analyzed the literatures. DW, WQ, and JY revised the manuscript before firstly submitted. HL, WY, and YL revised important intellectual content, added one figure, approved the final version to be published, and revised English grammar and sentences.

### Conflict of Interest

The authors declare that the research was conducted in the absence of any commercial or financial relationships that could be construed as a potential conflict of interest.
